# Comparison of the Conventional and Mechanochemical Syntheses of Cyclodextrin Derivatives

**DOI:** 10.3390/molecules28020467

**Published:** 2023-01-04

**Authors:** László Jicsinszky, Federica Rossi, Roberto Solarino, Giancarlo Cravotto

**Affiliations:** Department of Drug Science and Technology, University of Turin, 10125 Turin, Italy

**Keywords:** regioselectivity, energy efficiency, reaction mechanism, reaction kinetics, ball mill, ultrasound

## Abstract

Many scientists are working hard to find green alternatives to classical synthetic methods. Today, state-of-the-art ultrasonic and grinding techniques already drive the production of organic compounds on an industrial scale. The physicochemical and chemical behavior of cyclodextrins often differs from the typical properties of classic organic compounds and carbohydrates. The usually poor solubility and complexing properties of cyclodextrins can require special techniques. By eliminating or reducing the amount of solvent needed, green alternatives can reform classical synthetic methods, making them attractive for environmentally friendly production and the circular economy. The lack of energy-intensive synthetic and purification steps could transform currently inefficient processes into feasible methods. Mechanochemical reaction mechanisms are generally different from normal solution-chemistry mechanisms. The absence of a solvent and the presence of very high local temperatures for microseconds facilitate the synthesis of cyclodextrin derivatives that are impossible or difficult to produce under classical solution-chemistry conditions. Although mechanochemistry does not provide a general solution to all problems, several good examples show that this new technology can open up efficient synthetic pathways.

## 1. Introduction

The production of native cyclodextrins (CDs) is a fundamentally green process [1], as the waste can serve as raw material in the manufacturing of other products, e.g., industrial-alcohol production. However, this is seldom true for CD derivatizations because the production pipeline is often an energy-intensive process with environmentally unfriendly solvents. The most commonly used CD derivatives, such as (2-hydroxy)propyl- and (4-sulfo)butyl-βCDs (HPβCD and SBβCD), are produced on an industrial scale in a concentrated aqueous solution. However, the purification of the raw product and, especially, its conversion into a solid form is an energy-intensive step. Although water is an environmentally friendly solvent, the energy required in its removal can significantly increase manufacturing costs. The use of non-environmentally friendly solvents or the removal of water is also frequently necessary for the preparation of solid CD complexes or CD-containing nanoparticles. [2,3] Although the production of many intermediate products fails to meet the requirements of green chemistry, mainly because of solubility issues, some production steps may still comply with the principles of the circular economy.

Commercially available, widely used, industrially manufactured CD additives ((2-hydroxy)propylated, methylated, and (4-sulfo)butylated CDs) contain a mixture of a variably substituted compound in terms of the number and location of substituents. Three parameters, the residual unsubstituted CD, the degree of substitution (DS, the number of substituents on the macrocycle, as defined in the pharmacopeias), and the substitution pattern (isomeric composition of the derivative with the same DS and location of substituents on the glycopyranoside (Glc*p*) unit) characterize these CD derivatives. An alternative name for ‘randomly substituted’ is ‘statistically substituted’, since the arrangement of the substituents on a macrocycle is random. Randomness does not imply that there are no governing rules in a reaction, but the consecutive reactions usually occur at the (statistically) most likely reaction centers. In the case of a single chemical structure, the synthetic route to the product is generally irrelevant, but this is not the case for statistically substituted complex structures. Though the statistically substituted CD derivatives are robust, finding the most energy-efficient synthesis route resulting in an identical product is far from trivial. At the same time, higher drug-quality safety requirements mean that the production of chemically uniform CD derivatives also entails the development of more efficient production methods.

**Figure 1:**
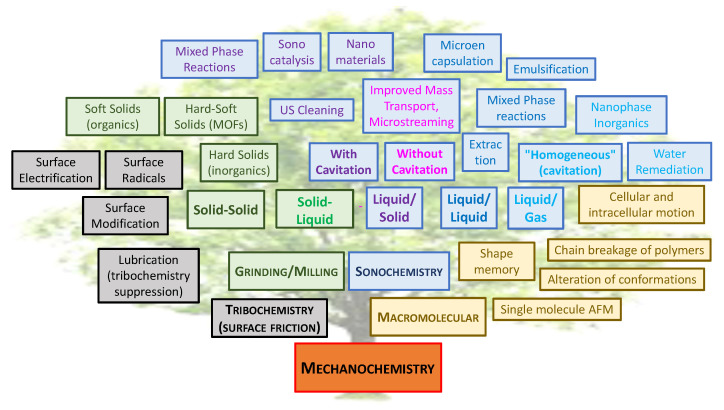
Tree of mechanochemical transformations, inspired by Suslick. [8] (AFM: Atomic Force Microscopy; MOF: Metal-Organic Framework).

A simultaneous combination of mechanical and chemical transformations always results in changes at the molecular level. This transformation, known as mechanochemistry, involves many physical, physicochemical, and chemical processes. [4,5,6,7] There are many ways to perform a mechanochemical reaction, like cavitation, shearing, friction, grinding, etc., see Figure 1 [8].

Though the occurrence of inorganic chemical reactions during grinding has been known for thousands of years, the utilization of the mechanochemical transformation was the child of the industrial revolution [9,10]. Ling published the first mechanochemical organic transformation at the end of the 19th century [11].

Cavitation only occurs in a liquid phase, in solutions or suspensions. During milling, many processes take place simultaneously. Reactions under comminution require at least one solid participant, and the presence of liquids—reagents, solvents, or even inert wetting agents—can significantly influence transformations. The energetics of reactions under cavitation and grinding show similarities, but the mechanisms are usually considerably different [12]. Reaction mechanisms under cavitation predominantly follow well-known classical solution mechanisms, whereas chemical transformations in grinding processes are fundamentally different [9,13,14,15,16]. In milling, when the liquid can dissolve one of the starting materials or product(s), the reaction mechanism becomes more complicated than in the simplified versions used in the modeling phase. In these cases, the reaction outcome is hard to predict. The water content of the reagents can often further complicate the situation. In the presence of (non-liquid) water, solids may stick to the reaction vessel, meaning that the formation of hard, rock-like solids may result in an incomplete reaction, as the grinding media can attack only the surface of the solids instead of crushing them. This effect can increase the bulk temperature by preventing heat transfer between the milled materials and the container. This often occurs when a shelf-dry CD is one of the starting materials. At present, there is little data available to predict a priori the feedback of the reaction system to the presence of water generated or already present [17,18,19,20]. In many cases, water is required not only for the dissociation of the inclusion complex, but also for its formation, and each new reaction is unique and requires preliminary experiments.

The thermal effect in solutions rarely results in high bulk temperatures. The collapse of micro/nanobubbles results in a short but extreme temperature effect. Although the temperature induced by cavitation is locally very high, it dissipates rapidly in the liquid not affected by cavitation [21].

The warming phenomenon of a ground reaction mixture is a complex mechanism. Besides the energy transfer between the grinding media and the reactants, combined with reaction heat, the heat-transfer rate also has a dominant role in the (average) bulk temperature. Local overheating is not unusual in the reactor because the mass of the colliding materials is significantly larger than the mass of the particles trapped between them. The limited heat-transfer rate rarely results in extreme temperatures, and the bulk temperature infrequently exceeds the melting points of the components [17,19,22].

While it is usually possible to visually monitor the physical changes in the reaction by cavitation, this is rarely possible during milling without dismantling the system. The only exception is reactions in a mortar, but limited reaction volumes and energy transfer make this method a technically peculiar case in syntheses. Although the preparation of complexes by kneading using mortars on a small scale is typical, no utilizable chemical transformations in kneading are currently known [23].

One of the most significant advantages of performing chemical reactions in ball mills is the absence of solvents. The missing solvents not only reduce the unwanted reagent consumption, for example, via hydrolysis but also allow the synthesis of compounds that are difficult or impossible to produce in solution because of the different reaction mechanisms. Of course, the lack of a solvent can eliminate the solvent effect on reactions. At the same time, omitting the high boiling point solvents commonly used in cyclodextrin reactions also allows less energy-intensive isolation of the (crude) product. In several cases, although water is an environmentally friendly solvent, its removal is often an energy-intensive or complicated procedure. Moreover, one beneficial feature of the ball mill method is its simple and fast reaction assembly and scalability [17,20,24,25,26,27].

Fritsch GmbH filed the first planetary ball mill patent in 1961. Since then, the instrument has undergone numerous extensions, including multi-purpose devices for parallel syntheses, and numberless new producers have appeared on the market. Their use as laboratory mills is now widespread as these ball mills are ideal for quick sample preparation and comminution with minimal loss. Today, a wide range of grinding media is available, enabling everyday applications in various fields, from simple sample preparation to chemical reactions and high-purity pharmaceutical procedures.

Ball mills are not only appropriate for the chemical or physical transformation of various materials, but manipulation in an inert atmosphere, mixing and homogenizing, and preparation of emulsions and creams are also possible.

Since grinding techniques have opened up a fundamentally new reaction pathway, this minireview focuses on the specifics of cyclodextrin derivatization in solution and ball mills. Ongoing developments in mechanochemical organic synthesis currently involve high-energy versions of grinding machines. Although their synthesis capacity is increasing daily, the production potential is still in the range of tens to hundreds of grams.

This minireview tries to summarize the recent advances in CD transformations and compare the differences and similarities between classical and mechanochemical CD derivatization. A short section briefly outlines the theoretical basis of mechanosynthesis to connect the theory and practice. A large number of publications do not allow the discussion of syntheses of all CD derivatives, so the authors have concentrated on the most common derivatives, which, after classical methods, have been attempted by ultrasonic or ball mill synthesis.

## 2. Discussion

The most reactive hydroxyls are the C(2)OHs. [28] Although for small reagents, C(3)OHs can show almost equal reactivities to C(2)OHs, C(3) hydroxyls are the less accessible ones on the macrocycle. Steric hindrance further reduces their reactivity when the neighboring O(2) is already substituted. In general, the primary hydroxyl groups of the glucopyranoside subunits are less reactive compared to C(2)OH. The primary OHs are not only the most flexible, but one substitution affects further substitutions significantly less, resulting in frequent uncontrolled substitution patterns. The higher reactivity of the secondary hydroxyls is related to the low acid dissociation constant (pKa ≈ 11.8–13) of these OH groups, [29,30], as the anion formed has a high nucleophilic potential. Although C(3)OHs can move away from the surface formed by the secondary hydroxyls during the continuous vibration of the pyranose ring, C(3)OH groups have a longer average residence time on this surface and are more hindered than C(2)OHs. [31] Since C(3)OH groups are only readily accessible to small reactants, only highly reactive species react with them rapidly before C(2)O substitution can occur and shield them. These factors result in lower apparent reactivity of C(3)OHs, which is significant when one of neighboring (on the same or previous unit) C(2)O is already substituted. In some cases, a complex with a suitable guest molecule in the solution [32,33,34,35] or solid [36] phase can react with these hydroxyls also, but these reactions are rarely appropriate for preparative purposes. [32] The more flexible primary hydroxyls tend to react when stoichiometric or steric factors reduce the availability of secondary hydroxyls or when the orientation of the complexed guest is suitable for reaction. [28]

Although the syntheses of many monosubstituted derivatives can be found in technical papers, their principal classification is based on the synthetic method. The CD derivatives prepared from an activated CD are often classified as second-generation derivatives, as the direct synthesis of a CD derivative is counted as the first generation.

The preparation of cyclodextrin complexes has used ultrasonication [37,38,39,40,41,42,43,44,45,46,47] or milling [48,49,50,51,52,53,54,55] for a long time. Mechanochemical methods are usually readily and quickly extendable technologies. Today, large-scale production, both in batch and flow reactors, can apply ultrasound. These techniques have a potential drawback, as metastable crystalline complexes may recrystallize during storage. Changes in the crystal structure can alter the physicochemical properties of complexes and reduce the benefits of complexation [56,57,58,59]. Sonication is advantageous for dissolution, dispersion, or crystallization, nevertheless, the formation of oxygen-, hydroxyl- and hydroperoxide radicals in an aqueous solution [60] can readily oxidize both the guest and host molecules. On the other hand, while the grinding media are usually chemically stable, their physical decomposition lends a disadvantage to the high-energy methods by product contamination. Using a grinding media with a higher Mohs hardness [61] can eliminate this weakness.

Studies of the ultrasound effects on chemical transformation have a significantly shorter history than the grinding-based technologies [62]. The first studies appeared at the end of the second decade of the 20th century [63]. In the early 1950s, Renaud published the first organometallic synthesis [64]. Mechanical and ultrasonic agitations can provide different reaction products, as was published almost thirty years later. Sonication of aqueous solutions of various natural gases showed the inorganic–organic formation of several prebiotic organic molecules [65], over and above the damage to several macromolecules such as DNA [66]. The evolution from these findings to the first utilization of carbohydrate derivatization was fast [67]. The penetration of synthetic sonochemistry into CD derivatization had already started a decade later [68,69]. The second decade of the 21st century saw the beginnings of CD application in ultrasonic transformations though the role of CD and the reaction mechanisms were unclear [70]. Although sonication could stabilize the interaction between CDs and salts, the primary purpose of ultrasound applications remains the production of nanoparticles [32,71,72,73]. Using ultrasound can significantly improve the synthesis of magnetic CD nanoparticles, though it is still a non-conventional CD complex and not a covalent association. Moreover, 6-monoamino-βCD can form a stable complex with magnetic nanoparticles [74]. Amino acid-modified βCD/CdSe/CdS-based quantum dots are also stable associations in hexane/water emulsions [44,46,75]. These complex materials remain stable for a long time under certain conditions, forming a transition between complexes and covalently/ionically bound compounds [76].

Using ultrasound [77] or grinding [78] in the syntheses of CD-pseudorotaxanes, which are also non-covalent CD derivatives, is common, even though, in these cases, the transformation of the macrocycle is usually not the goal.

The industrial application of grinding has a significantly longer history than that of organic manipulation. Whereas thousands of years passed between the first inorganic and organic syntheses, the mortar-to-ball-mill transfer of an organic synthesis required less than a century [10,11]. Grinding processes are frequently identified as solvent-free manipulation, although the presence of liquid phases in grinding is not unique. Inert solvents sometimes contribute as milling aids, either to help in obtaining smaller particles or to prevent the appearance of clumping effects. On the other hand, compounds with a low melting point can also form liquids, which can act as solvents in reactions.

Braun et al. revealed the elimination of the negative solvent effect in solvent-free reactions in the preparation of fullerene derivatives with the aid of γCD [79]. The mechanochemically triggered conversion also highlighted the role of complexation in solid-state transformations [80,81]. A collateral benefit of syntheses in ball mills is the ability to transform processes that would otherwise require polluting conditions into greener reactions. The first application of the ball mill in carbohydrate chemistry studied the classic Koenigs–Knorr glycosylation [82,83]. Similar systematic studies are rare in the production of alpha-glycosides. A clean S_N_2 mechanism is associated with high anomeric purity, but the presence of liquid reaction components does not allow concluding the pure solid-state processes. The exploitation of this stereoselectivity in glycoside synthesis is still sporadic.

In the first ball-mill reaction, (3-glycidoxypropyl)methyldiethoxysilane βCD was attached to silica, but the advantages of ball-milling over classical and microwave-heated reactions were not detailed [84]. The following year presented the first genuine CD-derivatization in click reactions using a planetary ball mill [85]. Optimization of the reaction conditions showed that ≈1.5 times faster rotation doubles the reaction rate, and 50 times scaling up did not significantly change the nearly quantitative yields. Menuel et al. reported the first natural cyclodextrin derivatizations on the secondary hydroxyl rim [18]. A further extension followed the pioneering works, using high-energy ball milling to synthesize randomly substituted CDs [19,26] and insoluble CD polymers [20,27]. Comparing the reaction products with those of classical solution methods also showed the limitations. While the mechanochemical reactions of CDs are simpler to set up and work up, yields and reproducibility are higher than in solutions, derivatization of activated CDs, and production of insoluble CD polymers have a higher potential. The statistical derivatizations of natural CDs showed significant differences in the substitution pattern of CDs [19]. However, statistical substitution is technically simple, but the complexity of the product composition in the solution often hinders reproducibility. Random derivatization is usually production-site dependent and difficult to reproduce by a third party. It is especially true when the apparent intellectual property protection further restricts the entry of competing manufacturers by various non-specific parameters [86].

The selective modification of a structure with multifunctional groups on a backbone is a more complicated task, unlike the random substitution of the same molecule. Since the first modification experiments, chemists have worked intensively to develop a feasible procedure for selective reactions on CD primary and secondary rims [87,88]. Mostly, these synthetic methods need organic solvents, energy-consuming work-up, and time-consuming purification. Large-scale production of regioselective substituted CDs is rare, and only two examples are known so far, the DIMEB [87] and octakis[6-(2-carboxyethylthio)-6-deoxy]-γ-cyclodextrin sodium salt (Sugammadex^®^). [89] Though it is also true that DIMEB means heptakis(2,6-di-*O*-methyl)-β-cyclodextrin, and the commercially available version is on sale as DIMEB, the heptakis(2,6-di-*O*-methyl) content of this product is usually 30–50% only. Another major challenge is that most armed and protected CD derivatives are the first steps only in a complicated synthesis.

### 2.1. Instruments

#### 2.1.1. Ultrasound

Although many ultrasonic (US) devices are available, most common laboratory cleaners are suitable for synthetic works. Common lab cleaners are monomodal devices, working in the 35–130 kHz range, and have rounded or cornered baths. Horn-type ultrasonic probes are flexible, and unlike ultrasonic baths, provide more control of the frequencies, energies, and reaction temperatures used. More recently, instruments in the 25–130 kHz range have become commercially available and are now accessible to routine synthetic laboratories, too. However, it is also true that flexible devices are significantly more expensive than traditional lab cleaners. The achievable particle sizes in these devices depend on both the applied frequency and energy. Higher frequency/energy is better for the preparation of micro- or nanoparticles [90], but the situation is not so clear for chemical reactions [91]. The size of the cavitation bubbles varies inversely with energy and frequency, which can significantly affect chemical reactions in aqueous solutions [92]. The selection of the most appropriate instrument depends on the actual project [60]. Ultrasonic peak power is usually three to four times higher than the effective power, which, for laboratory cleaners, is between 100 and 400 W. Although modern devices have temperature control, this function is inappropriate for controlling the reaction temperature. However, ultrasound-triggered cavitation provides high temperatures in the microenvironment during the collision of bubbles, and that temperature shows less dependency on bulk temperature [8,21]. In most laboratories, ultrasonic baths are for cleaning, degassing, or facilitating dissolution, but rarely for chemical reactions under controlled conditions. While stirring inhibits cavitation, it can help in the dilution near the proximities of cracked insoluble but can prevent a chemical reaction.

#### 2.1.2. Ball Mills

There are many types of grinding machines, such as rod-, ball-, and attrition mills. The first ball mill was a further development of a rod mill, which appeared at the end of the 19th century in the ore and cement industry [93]. The geometry of ball mills is usually cylindrical, in which centrifugal force and gravity during rotation or shaking control the movement of the balls. Through collisions, the kinetic energy can not only shred larger particles but can convert mechanical energy into the activation energy needed for chemical reactions. The construction materials of balls and cylinders, by lining, show a wide variety from steel to natural and artificial compounds, like ceramics, zirconium oxide, or plastics. The variable hardness and durability of the materials allow using ball mills in many industrial applications, including the high-purity technologies required in the pharmaceutical industry. By the middle of the 20th century, inorganic mechanochemical chemical reactions had become widespread on an industrial scale. By the middle of the 20th century, inorganic mechanochemical chemical reactions had become widespread on an industrial scale. The emergence of new materials and technologies accelerated the development of more energy-efficient tools. The advent of high-energy ball mills slowly led to the development of mechanochemical organic syntheses, and the ball mill technology started to alternate the mortar-based methods [9,94,95].

#### 2.1.3. Comparison of Planetary and Vibrating Ball Mills

The most popular grinding instruments are currently vibrating and planetary ball mills. Although both instrumentations are suitable for many chemical transformations, the vibrating version usually operates on a smaller reaction scale. The compatibility of the different ball mills in chemical reactions is unclear. In the case of a heterogeneous reaction, the ball mill version shows significantly higher efficiency [17]. In a classical S_N_2 test transformation, the Br→I exchange of benzyl bromide showed only 40% conversion after 16 h grinding [96], whereas the reaction in the planetary ball mill produced 75–85% conversion within half an hour. The tosyl group was stable under the planetary ball mill treatment if no nucleophile was present in the system. Studies on the exchange rate of the tosyl group appended to a primary hydroxyl of βCD in the presence of various nucleophiles did not show direct relationships between the Lewis hardness of the nucleophile, as was previously supposed. This reaction showed some cation dependency [17]. With the addition of a strong base during grinding, a 3,6-monoanhydro unit formed, like in the solution cases [18]. The direct synthesis of a 2,3-mannoepoxy unit containing macrocycle is also possible from the 2-O-monotosylated CDs, using alkali hydroxides. The formation of the 2-O-monotosylated CDs is very fast in a vibrating ball mill utilizing the favorable conformation of tosylimidazol/CD complexes. Manipulation in a vibrating mill resulted in 2-O-monotosylation in all studied CDs using Na, K, Cs, and Rb carbonates since the sodium bicarbonate and Li_2_CO_3_ were inappropriate to activate the C(2)OHs. The substitution efficiency showed cation dependency, and the residual/formed water may also provide some acceleration effects.

### 2.2. Reaction Mechanism

In most solution reactions, molecularly dispersed reagents react, unlike in the ball mill-conducted version. The ultrasound-assisted mechanochemical reactions work in solutions or suspensions and usually follow well-known reaction kinetic models. In these reactions, only energy transfer differs from the conventional methods. If one or both components are poorly soluble in a given solvent, the reaction occurs in solution or on the surface(s) of the reagents. In entirely homogeneous or partially heterogeneous reactions, cavitation provides a high-energy transfer to the microenvironment. In many cases, solubility cannot only be increased via the fragmentation of the poorly or insoluble reagent, but the large molar excess of the dissolved reagent also ensures faster reactions. In ultrasonic reactions, temperature control is possible, unlike in ball mill reactions. The advantages of ultrasonic activation have been successful in the preparation of per-6-aminoalkyl-cyclodextrins [97].

**Figure 2:**
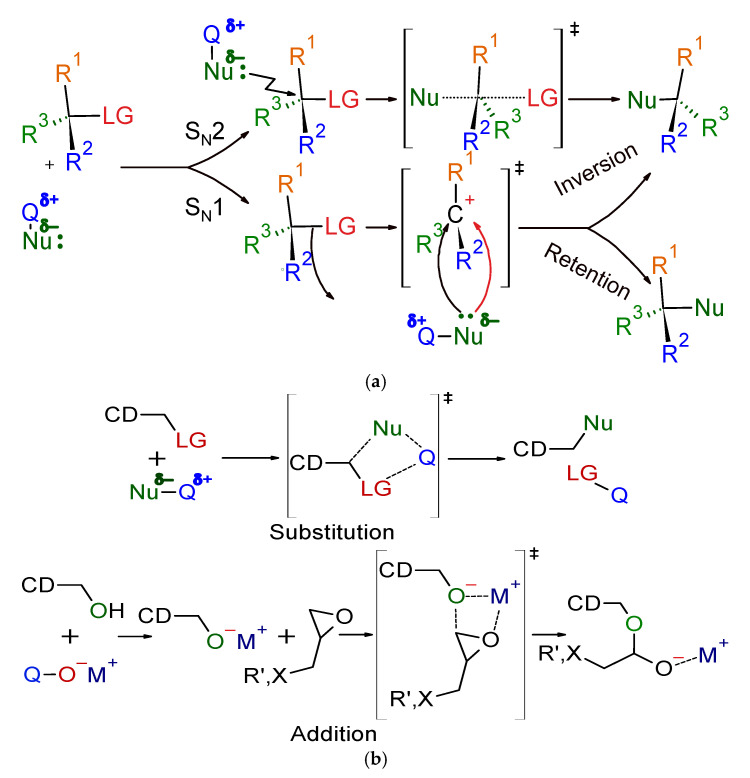
Simplified reaction mechanisms of nucleophilic reactions in solution and a ball mill. (**a**) Nucleophilic substitution in solution; (**b**) Nucleophilic reaction of cyclodextrins in a ball mill; CD: Cyclodextrin; LG: Leaving Group; M: Metal; Nu: Nucleophilic moiety; Q, R’: organic group or hydrogen.

In solution, the substitution/addition reactions follow the simplified classic mechanisms, and the solvents often can influence not only the reactivity but also the S_N_-type can change. In grinding reactions, the reactions most often go as a four-centered concerted reaction. Of course, the reaction mechanisms are not textbook-like black and white for either method, as seen in Figure 2. Unlike solution reactions, the restricted flexibility of the molecules means that internal substitution reactions (S_N_i) rarely occur in the solid state. While, in the solid-state, intermolecular S_N_2 reactions depend on the contacts of interacting particles only, the S_N_i reactions may show more structure-dependent behavior, owing to the solid-state structure, which can be either favorable or unfavorable. An example is the formation of 3,6- and 2,3-monoanhydro-CDs [17,18].

**Figure 3:**
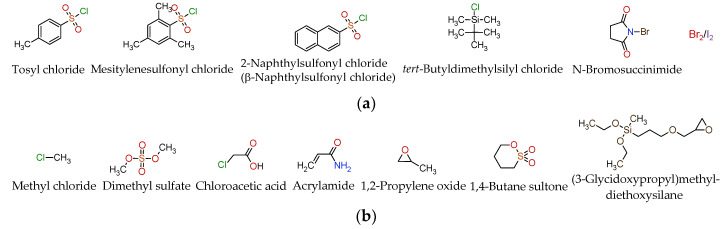

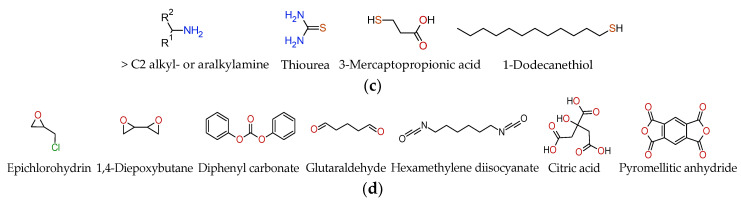
The most common reagents used in CD syntheses. (**a**) Reagents for regioselective mono- or oligosubstitutions; (**b**) Reagents used in random substitutions; (**c**) Reagents for regioselective persubstituted CDs; (**d**) Crosslinking reagents used for CD polymerization.

The most common reagents used to synthesize CD derivatives are shown in Figure 3. Of the chemicals shown, methyl chloride, 1,2-propylene oxide, and 1,4-butane sultone are used in the several hundred kilograms to ton-scale production of βCD derivatives, while tosyl chloride and dimethyl sulfate are for the several kilograms scale CD derivatization. In the kg-scale preparation of Sugammadex^®^, 3-mercaptopropionic acid reacts with octakis(6-deoxy-6-halogeno)-γCD. Epichlorohydrin is the most common crosslinking agent for soluble and insoluble CD polymers. Other reagents are mostly limited to the experimental laboratory scale and rarely exceed ten to one hundred gram production batches.

### 2.3. Reaction Kinetics and Energetics

Many, more or less reliable, reaction kinetics models have been created and tested in the last half century, but this task is inherently more complex than in solution. In the solid state, more processes proceed simultaneously. Cracking, attrition, shearing, etc., are all independent events, and any liquid phase(s) can further complicate the situation. Molecularly dispersed reagents react in most solution reactions, unlike in reactions conducted in ball mills.

Although Griffith had already sketched and verified a one-dimensional model of fractures on a molecular basis—“theorem of minimum potential energy”—in the early 20th century [98], its extension to a 3D spatial model was unsuccessful until the late 1940s [99,100]. The original model explained many physicochemical properties in crystals but was less successful in the case of non-crystalline elastic materials.

An extension of the basic concepts to 3D models did not show significant differences from experimental results. The appearance of computers accelerated the refinement of the old empirical models. It was clear from the beginning that Young modulus, Poisson’s ratio [101], specific surface energy [98], and tensile strength [102] play a crucial role in size reduction. Later, further refinement of the models made it necessary to take into account more parameters, such as the number of internal defects in solids, the dissociation energy of bonds, etc. Mathematics and the physics of fractals have greatly improved the tools for fracture kinetic modeling.

The temperature in the ground system is not only not constant, but usually impossible to measure. As known from the Arrhenius equation, a ten-degree change in reaction temperature provides almost threefold changes in reaction rate. In a ball mill, the solid bulk and local temperatures are not necessarily equal and are usually different from the ball temperature. Temperature control is rarely possible compared to solution reactions. Reactions proceed on the surface of the reactants between the colliding balls, and these complications allow for the establishment of empirical relationships only, even for two-component systems. Although some sequential addition is also possible in ball mill reactions, an “infinite” excess of one of the reagents produced in solution reactions by dropwise addition is impossible in this case.

Although modeling both the fragmenting kinetics and the breaking/formation of bonds is more or less consistent, contact surface formation, their areas, and the energy transfer between the colliding surfaces entail empirical approximations. The kinetics become even more complex if the system is not a simple A→B, A+B→C, or A+B→C+D reaction, as in the case of multiple substitutions of cyclodextrins.

Different theoretical approximations have been developed for reactions of reduced complexity, as shown in Table 1. A detailed discussion can be found in a recent review by Alrbaihat et al. [103].

Autocatalytic reactions during grinding show similar sigmoidal kinetics as in the solution [108]. This kinetic behavior is related to a positive feedback mechanism where a catalyst, such as water, is formed as the reaction proceeds [109].

Takacs attempted to describe the complicated energetic situation of heat transfer to the jar wall and balls [110]. The formulated equation contains many hard-to-calculate parameters in the following second-order differential equation:

d^2^Θ/d(r/x)^2^ + (k·r/x)·dΘ/d(r/x) = −Θ (Q·E·r^2^·c·a·e^(−E/R·T0)^)/(λ·R·T_0_^2^)

where Θ = (E/(R·T_0_^2^)) (T−T_0_), k, x, r and λ are geometric parameters (k = 0:plane, k = 1:cylindrical, and k = 2:spherical jar), E is the activation energy, R the gas constant, T the temperature of the medium in K, T_0_ the temperature of the wall in K, Q is the reaction heat, c is the concentration of the reactants, and a is the frequency factor of the Arrhenius law.

Although the theoretical basis for mechanochemical syntheses appears to be sound, the computational difficulties and many empirical parameters involved mean that an a priori prediction of the outcome of organic reactions is rarely possible.

### 2.4. Monosubstitution

Efficient monosubstitution is usually a difficult task on CDs. The reactivity of the three types of hydroxyls is not equivalent, and the bulkiness of the reagents, complexation properties, and solvent effect can also significantly influence the substitution paths. Monosulfonyl CD esters are the most commonly utilized of the first-generation monofunctionalized CD-derivative compounds as they are good intermediates for several, principally chiral, selectors. Pyridine is a favored solvent in classic syntheses; however, in the case of cyclodextrins, the product is not always uniform, and multiple substitutions usually also occur [111]. Although C(2/3)O-sulfonates can serve as reactive intermediates in supramolecular structures, after the configuration change on the incriminated C(2/3), the product no longer contains only Glc*p* units. The secondary aryl sulfonyls are easily attacked by the neighbor O(2/3) hydroxyls forming a manno- or alloepoxides or 2A,3B-anhydro cyclic carbohydrates [112], although the regeneration of the gluco-configuration is also possible by the formation of an interglucosidic 1,4-dioxane ring [113]. Methanesulfonylation rarely results in monosulfonyl esters due to the small and reactive species. The bulkier tosyl group seems more appropriate for monosubstitution [114], but the reaction shows a strong solvent effect [111,115,116,117]. Although mesitylene- [118,119] and naphthylsulfonyl [120] esters are also suitable leaving groups, their production costs are higher and the regioselectivity of the reagents used is minimal [120,121,122].

Ultrasound successfully accelerates the synthesis of 6-monotosylated α/β/γCDs in a concentrated solution of CDs and tosyl imidazole [123].

In a ball mill, in the absence of a solvent, the selective O(2) tosylation and 6-O-mesitylenesulfonation is possible, but O(2) substitution causes a conformation change on a secondary carbon and removes the formed derivative from cyclodextrins [18,124]. The particle size and crystallinity of the reagents can significantly influence reaction outcome and substitution pattern. In addition, the longer reaction times in a vibrating ball mill favored the formation of polysulfonates. In conclusion, the mechanochemical synthesis of monoderivatized CDs on the secondary hydroxyl rim can be efficient if the reactive guest is favorably complexed in the macrocycle’s cavity.

Due to the potential hydrolytic side reaction, (trans)esterification reactions can be challenging in solution. Some immobilized enzyme-catalyzed (trans)esterifications have successfully utilized ultrasound to facilitate substrate access to the active site of the lipase enzyme [125]. The reaction proceeded on the primary hydroxyl of the CDs and monosubstitution only occurred on αCD, while increasing macrocycle size had an effect, with one and two methotrexates being attached to β- and γCDs, respectively.

Unlike esterification, mechanochemistry showed more advantages in monoalkylation reactions. Strong bases can ionize in solution C(2)OHs, which have the lowest pKa values among the CD hydroxyls [28,126], allowing a regioselective substitution. However, the reactions are rarely quantitative, and many byproducts can appear. Non-conventional activation can either accelerate these reactions by increasing contacts between the reagents (ultrasound) or via the accelerated heating of the reaction mixture (microwave (MW)). Although two non-conventional reaction conditions, MW or US irradiation, failed to improve the yield between the reaction of methyl (3-bromopropyl)-2-iodobenzoate and βCD, the irradiations dramatically reduced reaction time from three days to four hours (US) and one hour (MW) [127].

While direct mechanochemical CD derivatization does not generally offer a green alternative to classical methods, the reaction of activated CDs with nucleophilic reagents possesses significantly higher potential in rendering chemistry greener. The use of ultrasonics for second-generation CD syntheses does not generally offer many advantages because of solvent demand. Grinding technology’s biggest advantage is solvent elimination, which reduces potential side reactions and makes workup economically attractive. For the time being, organic syntheses use high-energy ball mills that still somewhat limit the synthetic scale, and there have been no attempts yet to use classical ball mills in those transformations.

Some inhomogeneous reactions can exploit the combination of the fast warming of MW with the effective attrition of ultrasound. 1,3-dipolar cycloaddition is a good target for both benefits as at least one component is often poorly soluble in the solvent used. The Cu(I) salts are almost insoluble in water and organic solvents, and neither are lipophilic organic substrates in water. The combined technique effectively activates the insoluble copper powder, triggering a click reaction between cyanine dyes and 6-monoazido-6-monodeoxy-βCD [128].

Reduction of 6-monoazido-βCD to an amine using a Pd/C catalyst and H_2_-gas is difficult because of the poor solubilities of both the starting compound and product. Although the ultrasonic treatment of the double/triple heterogeneous reaction mixture accelerated the hydrogenation [129], the efficiency was far below the classic method [130].

Sulfonyl ester CDs are good intermediates, but the replacement of the sulfonyl group to the most common azido function or a heteroatom, like S [114] or Se [131], shows a solvent effect. Hydrolysis occurs in water [132] and *N*,*N*-dimethyl substitution occurs in DMF [133], while in DMSO, oxidation [134] can occur that reduces the yield, and, many times, the byproducts are hard to remove.

The synthesis of mono(alkylamino)-CDs from monotosyl intermediates has not yet been reported, although sonication could be possible if the reagent is a liquid amine.

Ball milling does not use solvents, and the absence of a solvent can reduce the amounts of necessary reagents and improve yields. Systematic studies on nucleophilic replacement on 6-O-monotosyl-βCD [17] showed a weak correlation to the theoretical reactivities based on the Lewis acid-base theory [135].

In summary, the mechanochemical mono-derivatization of the secondary hydroxyl rim can be more effective than in a solution reaction if the reactive guest forms a spatially favorable complex. The alternative synthesis of the most common CD derivatives prepared from the O(6)-arylsulfonylated intermediate is possible using a green preparative method.

### 2.5. Regioselective Multiple Substitutions

The restricted movement of reagents means that the selective multisubstitution of natural CDs prefers solution reactions. Sonication is a reasonable choice in some cases as it can exploit the solvent effect, steric hindrance, and reagent solubilities. Technically, several multifunctional reagents also fall into this category, and diactivated reagents can react with another hydroxyl group on the identical macrocycle. Sterically suitable multifunctional reagents in a solution can easily access the corresponding hydroxyl group(s). As their reactive centers are relatively far from each other, macrocycle flexibility can lead to an appropriate conformation for a favorable reaction. This is usually impossible in the solid state, mainly when the reagent is a good guest and complex formation further prevents reaction. The potential low solubility of the reagent can be a further advantage in an identical CD, as internal reactions in diluted solutions are more probable.

Ultrasound can restore the reactivity of blocked C(3)OH groups, although the regioselectivity is evidently independent of sonication. A typical example is the synthesis of various per-2,3-O-alkyl-6-TBDMS-βCDs in DMF, and ultrasound helps the reaction on the most hindered oxygen atoms of a CD [136,137].

Mechanochemical synthesis with an already activated CD also has several advantages in regioselective multiple substitutions. The preparation of selectively activated CD derivatives via classical synthetic routes is often expensive, as they usually require complex purification or have limited availability. The efficient use of costly reagents is more than demand, and, in these cases, mechanochemistry cannot only make the production greener but also significantly reduce costs. The most common armed CDs are only soluble in high boiling solvents, which are difficult or almost impossible to remove entirely due to complex formation. Ultrasonic treatment can often significantly improve yield and purity if the reagents are insoluble in the reaction medium. The frequent opening and closing of the reaction vessel during the spoonwise addition of a solid component introduce additional critical points to reproducibility, especially when the reaction temperature differs from room temperature. Sonication in the preparation of per-6-aminoalkyl derivatives significantly increased the reaction rate in neat alkylamines at moderate temperatures. The poorly soluble per-6-activated CDs slowly dissolve in the liquid, and the enormous molar excess of the amine allows the complete replacement of the halogens to occur, minimizing potential 3,6-anhydroglucopyranoside-forming side reactions [97].

A direct reaction between an oleate epoxide and CDs in a ball mill resulted in multisubstituted derivatives, and, although it could be somewhat regioselective, the publication failed to discuss the substitution pattern [26].

The exhaustive substitution of multi-activated CDs in solution is not self-evident. The resulting by-products have similar physicochemical properties to the target derivative, which makes the purification process costly. Sugammadex^®^ has slowly become a standard protocol in the post-operative recovery of patients and, as an active substance, requires high purity and a known impurity profile. The conventional synthesis of Sugammadex uses NaH, per-6-bromo-γCD, 3-mercaptopropionic acid, and DMF [138]. Although the procedure appears straightforward, many side reactions can occur, such as 3,6-anhydroglucopyranoside units, disulfide bridges formed during the retro-Michael reaction, and the substitution of halides with dimethylamino groups. A systematic study in a planetary ball mill on the preparation of per-6-derivatized CDs showed the advantages of solvent-free conditions. The reaction in a ball mill uses the significantly less dangerous potassium *tert*-butylate instead of NaH, a smaller molar ratio of 3-mercapto propionic acid than the solution reaction, and the absence of the pollutant *N*,*N*-dimethylformamide also eliminates typical side reactions in solution. Crude product processing is also straightforward because the destruction of NaH excess and removal of the mineral oil (from the NaH) is unnecessary. The raw product is easily soluble in water under safe conditions, and at low pH, the product crystallizes [139].

However, complex formation in ball milling reactions can be detrimental and reduce the reaction rate. The preparation of per-6-azido CDs in a ball mill has shown that the differences in complexation between NaBr, NaI, and NaN_3_ significantly affect the substitution yield. The synthesis of per-6-azido-CDs from 6-halogenated CDs in solution is complicated because the required amount of NaN_3_ is not soluble in organic solvents and its stepwise addition to the reaction mixture is necessary. The long reaction time at high temperatures results in moderate yields, not to mention the difficulties in solvent removal. In solution, per-6-iodo-CDs may be more efficient as the solution reaction follows the usual S_N_2 scheme. However, iodinated derivatives are also more likely to undergo undesirable transformations. In solution, particularly in DMF solutions, the NaI complex formed can dissociate readily, and the free rotation of the activated CH_2_ groups also provides an additional accessible reaction site. In the absence of solvent, NaI diffuses slowly out of the cavity. Although more sodium azide can help to displace the formed NaI from the CD cavity, iodides are better complexing guests than azides.

The order of reactivity of organic halogens in the solid state is usually the reverse of that in solution, but per-6-chloro-CDs were unsuitable for synthetic purposes, as they could not react in both solution and solid phases [139].

### 2.6. Multiple Random Substitutions

Randomly (or statistically) substituted CDs occupy a marked position among the CD derivatives. Currently, three of these derivatives are on the market in industrial quantities: methylated βCD (RAMEB), (2-hydroxy)propylated β- and γCDs (HPβCD and HPγCD), and (4-sulfo)butylated βCD sodium salt (SBβCD). Among them, the (2-hydroxy)propylated and the (4-sulfo)butylated versions are approved as drug excipients, as well. Although their derivatization is technically simple in principle, in practice, only the (2-hydroxy)propylated version exhibits a DS and substitution pattern broad enough to allow product preparation by green chemical processes [19,20,27,140]. Other derivatives commonly used in analytical chemistry are carboxymethylated and -ethylated derivatives and methylated 6-monoamino-βCDs. The applications require small quantities, so their production on an industrial scale is generally not feasible, with annual requirements rarely exceeding 100 g. The preparation of RAMEB is, itself, the result of a greenwashing process, and the alkylating agent used is unsuitable for synthesis in either an ultrasonic or milling environment. For similar reasons, the preparation of per- and randomly methylated 6-amino-βCD is only partially appropriate for greenwashing. As shown above, the synthesis of 6-amino-βCD involves at least one suitable green step to eliminate the unfriendly organic solvent from the production pipeline. Although this simplifies and speeds up the laboratory works only.

A systematic study has shown that substitution patterns in planetary ball mill syntheses can be significantly different from products obtained under classic reaction conditions [20,141]. This significant difference is not surprising, considering the peculiarities of the reaction setup and the fact that the reactants collide randomly. Although the preparation of (2-hydroxy)propylated γCD with high DS in a ball mill can significantly reduce the number of synthetic steps, this is only possible because the substitution pattern is roughly defined. Epoxides are generally highly reactive species. Furthermore, propylene oxide is a low-boiling liquid. Therefore, the reaction assembly needs specific, but not extreme handling: short milling of CDs with the necessary NaOH or OH forms the alkaline salt of CDs, and the addition of the epoxide requires intensive cooling of the reaction mixture and vessel, or else the epoxide reacts fast, and the exothermic reaction destroys the reagent instead of CD derivatization.

The highly stringent DS distribution pattern of (4-sulfo)butyl-βCD is patented [86]. Proving that the green alternative has identical complexation, pharmacological, and biological properties compared to the originator’s compound mixture is expensive.

In the case of derivatives used in analytical chemistry, where separation methods are constantly evolving, the chances of success seem high. For these derivatives, the high reproducibility of the milling techniques and the elimination of product dependence on the laboratory and the synthesizer could be the best advantage.

### 2.7. Polymeric Cyclodextrin Derivatives

Although many CD-based nanoparticles contain both polymers and derivatized CDs, their preparation utilizes ultrasound, and in minority ball mills, they are dominantly not CD polymers [142]. There are two essential groups of CD-derived polymers. If the polymer contains CD-monomer units, i.e., several macrocycles covalently linked to a polymer backbone, the compound is not a CD polymer (CDP) in the classical sense. Sometimes CD-based stable metal-organic frameworks (MOFs) can also be CD-containing polymers, but fundamentally, the attractive forces between CDs—whether polymers or monomers—and metal oxides are generally non-covalent. In these composites, the unique CD structure lends itself to strong interactions between the metal-oxide interface and the CD. The preparation of MOFs routinely uses ultrasound or mills [76,143,144,145,146,147]. CDPs contain CDs and linkers, which, regardless of the type of reactions, (poly)addition, (poly)condensation, or polymerization, used to prepare them, form the CD polymer. CD polymers are usually CD derivatives that contain linkers connected with ether linkages to the CDs [148] or compounds polymerized from CDs containing double bond substitution [149,150,151]. Although, many times, the term is a synonym of nanosponges, as well. If any ester, amide, or urea-like covalent bond characterizes the polymer, the product is called a CD nanosponge [152,153]. The fundamental difference between the two polymer types is that the ether-like bond is chemically more stable. Both variants can be soluble (CDPS) and insoluble (CDPIS). Insoluble polymers are definitely insoluble, and aggressive solvents can only dissolve them by disrupting the polymer or macrocyclic structure.

Although CDPs have been the focus of continuous developments since the mid-1960s, their utilization in various daily applications is uncommon. The principal problem is that the syntheses show strong laboratory and personnel dependency. Although the potential parenteral pharmaceutical use of soluble CDPs, despite their favorable properties, is not expected, as their structural characterization for medical use does not fit all the approval criteria, of course, there are many other application fields. However, CDPIS have a higher practical potential than soluble versions, as they are unsuitable for parenteral administration and do not penetrate through the skin or mucosa. This feature could simplify the registration process and eliminate many pharmacokinetic and pharmacodynamic concerns.

Using an ultrasonic treatment in CPDIS preparation is broad [154], not in terms of a chemical reaction, but the production of small particles. Though the fabrication of small particles can use ultrasound, the high amount of solvents does not make the process green. Sonicating a hydrophilic epichlorohydrin CD polymer suspension could also significantly improve the adhesion to fused silica used in GC [155].

Chemical reaction occurring under extrusion conditions is a discovery and has become the subject of current studies. Extrusion is a well-known technique in various material technologies, with complex hybrid physics, and combines shear, cavitation, and sometimes torrefaction phenomena [156,157,158]. Extrusion processes with chemical transformation are promising techniques for various CD composites [159]. The combination of hot extrusion and ultrasound is another innovation of the 21st century [160,161].

Isolation of insoluble polymers prepared in solution does not always easily dispersible polymer. On the other hand, in an aqueous reaction mixture, the hydrolysis of the reagent results in a more hydrophilic product by the dihydroxy side chain. A multi-functionalized reagent can react with the already substituted but hydrolyzed side chains, resulting in a higher distance between the CD units. Although the solution polymerization of a complex by the preformed complex can provide a suitable conformation for a more effective complexation property to the CDP, both the guest and complex need to fit some requirements. Unfortunately, the imprinting version of the polymerization is restricted significantly by the guest properties. The solution method is more common, but a higher potential of the solid-state synthesis can be favorable because, unlike in solution, the complexes are more stable in the absence of solvent used in the polymerization reactions.

The solid-state polymerization, by the more effective utilization of the multifunctional reagent, can result in a more compact polymer in which the CD units are closer to each other. The spatial proximity of the CD units can be more favorable for guest molecules where the distance between the complexable moieties is close together [20,27].

The preparation of nanosponges generally requires the use of various high-boiling solvents. Removal of the decomposed and unreacted crosslinkers and the solvent are challenging. The mechanochemically prepared nanosponges mainly showed at least as good complexation properties as those prepared under classical organic conditions [129,162,163,164].

## 3. Conclusions

In ultrasound-assisted synthesis, the greatest potential lies in reactions in which at least one essential component is poorly soluble or insoluble under the conditions used. Mechanochemical methods are valid synthetic alternatives to the most common CD derivatives from the O(6)-arylsulfonylated intermediate that may get rid of toxic solvents. Nevertheless, a regioselective multisubstitution is still a long way off. Randomly substituted derivatives can often be conveniently prepared in ball mills, though the substitution pattern may differ from the product obtained in classical reactions. CD-based polymers are excellent targets for mechanosynthesis, showing a more favorable reagent utilization.

## Figures and Tables

**Table 1:** Reaction-kinetics models of ball milling in the A + B → C reaction.

Model	Formula	Legend	Used For	Literature
Simple	dc/dt = −k∙c^n^	n: reaction order (not necessarily integer) n = 0.5 Phase boundary controlled reaction (contracting area) n = 0.67 Phase boundary controlled reaction (contracting volume) E_T_: activation energy	kaolinite amorphization	[103,104]
Probability	v = −K_m_∙χ∙S_max_(1−e^−k∙t^)	v: reaction rate, K_m_: the probability of reaction, χ: contacting probability of particles in collisions, S_max_: contacting area of A and B components collisions	phenomenological rate law calculations	[105]
Interatomic bond breakage (Zhurkov)	K = k_0_∙e^−((E0−∆E)/R∙T)^	k_0_: bond dissociation constant; E_0_ bond dissociation energy, ∆E: activation energy reduction when bond dissociated without stress		[106]
Boltzmann distribution (Basedow)	K_s_ = k_S0_∙e^−(Es/a∙J)^	k_s0_: shear-induced degradation constant, E_s_: shear activation energy, J: input rate is, a: average amount of mechanical energy in each bond in a sheared system	degradation of polymeric chains	[107]

Where the meaning of c: concentration (weight fraction) of a component; t: time; k: reaction rate constant; R: gas constant; T: temperature in K.

## Data Availability

Not applicable.
